# Effects of Steady Flow on Magnetoacoustic-Gravity Surface Waves: I. The Weak Field Case

**DOI:** 10.1007/s11207-017-1051-1

**Published:** 2017-01-19

**Authors:** R. Erdélyi, J. F. Mather

**Affiliations:** Solar Physics and Space Plasma Research Centre (SP2RC), School of Mathematics and Statistics, University of Sheffield, Hicks Building, Hounsfield Road, Sheffield, S3 7RH UK

**Keywords:** Flow, Dynamics, Waves, Magnetic fields, Kelvin–Helmholtz

## Abstract

Magnetoacoustic gravity (MAG) waves have been studied for some time. In this article, we investigate the effect that a shear flow at a tangential discontinuity embedded in a gravitationally stratified and magnetised plasma has on MAG surface waves. The dispersion relation found is algebraically analogous to the relation of the non-flow cases obtained by Miles and Roberts (*Solar Phys.* **141**, 205, 1992), except for the introduction of a Doppler-shifted frequency for the eigenvalue. This feature, however, introduces rather interesting physics, including the asymmetric presence of forward- and backward-propagating surface waves. We find that increasing the equilibrium flow speed leads to a shift in the permitted regions of propagation for surface waves. For most wave number combinations this leads to the fast mode being completely removed, as well as more limited phase speed regimes for slow-mode propagation. We also find that upon increasing the flow, the phase speeds of the backward propagating waves are increased. Eventually, at high enough flow speeds, the wave’s direction of propagation is reversed and is in the positive direction. However, the phase speed of the forward-propagating wave remains mainly the same. For strong enough flows we find that the Kelvin–Helmholtz instability can also occur when the forward- and backward-propagating modes couple.

## Introduction

Wave phenomena that can be modelled as surface waves are ubiquitous in nature. They may occur when a medium changes properties at an interface, with perturbations of largest amplitude at the interface (surface) and perturbations decaying away from the surface. In a magnetohydrodynamic (MHD) sense, the change in magnetic field (or other properties *e.g.* density, temperature, *etc.*) of a plasma at an interface can lead to perturbations propagating along the interface in the form of magnetoacoustic surface waves (Roberts, 1981a,b). If the plasma is embedded in a gravitational field (perpendicular to the magnetic field), magnetoacoustic-gravity (MAG) surface waves can occur (Miles and Roberts, 1992). These surface waves are of considerable interest in a solar context *e.g.* for coronal heating or oscillations in sunspot penumbrae (see *e.g.* Giovanelli, 1972 or for more recent studies Freij *et al.*, 2014).

Many plasmas in nature, however, do not exhibit static equilibria, and for the most part, are dynamical; strong background equilibrium flows may occur. For example, the interior of the Sun is highly dynamical, exhibiting large-scale flows, *e.g.* differential rotation; close to the surface, meridional flows; and small-scale flows of the surface *e.g.* penumbral flows (Evershed flow). As a next step, it is therefore sensible to study magnetoacoustic surface waves in such media considering the effect of flow. The addition of a uniform steady flow in a uniform and infinite medium will just lead to the Doppler shift of the wave frequencies. However, any variations in the medium (*e.g.* discontinuities or smooth variations) or in the steady flow affect the surface wave propagation to a greater and more complex extent.

In this study, we investigate the effect of a steady flow at a tangential discontinuity. The equilibrium plasma is similar to that given in Miles and Roberts (1992), but with a steady flow added to the bottom layer. This configuration may represent a simple view of large-scale convection or meridional flows affecting surface MHD waves that are present at the interface of the solar interior-atmosphere. A shear flow at a tangential discontinuity can be of importance in the stability of surfaces in hydrodynamic and MHD theory. A shear flow can lead to the Kelvin–Helmholtz instability: when the shear in flow is great enough, a forward- and backward-propagating wave couple together, causing the instability (Chandrasekhar, 1961). The negative-energy wave instability (Ryutova, 1988, Ruderman and Goossens, 1995, Ruderman and Wright, 1998, Tirry *et al.*, 1998, Taroyan and Erdélyi, 2002) can occur for lower flow shears than this, however, making it an interesting area of study. This instability is intimately linked to the so-called dissipative instability (Ruderman *et al.*, 1996, Erdélyi and Taroyan, 2003), where the “negative energy” (*i.e.* energy loss) is due to some form of dissipation being present in the system.

At a tangential discontinuity the flow can affect which magnetoacoustic waves are able to propagate, as the cutoffs, determining the propagation window (*i.e.* changes from non-leaky to leaky modes), can be shifted. This was demonstrated in Nakariakov and Roberts (1995) and Terra-Homem, Erdélyi, and Ballai (2003). For a certain range of flow parameters, the fast wave in a magnetic slab could not propagate. Furthermore, in general, the phase speed of a wave propagating in the direction of the flow is increased to a small extent by the bulk motion (in comparison to the same wave in the static medium), whereas a wave propagating in the counter-flow direction would be decelerated to a much greater extent. If the flow is strong enough, then the phase speed of this latter so-called backward-propagating wave can be increased enough for the direction of its propagation to change. In terms of the stability of a tangential discontinuity, this change in direction of the propagation can be of great importance, *e.g.* leading to the phenomenon of negative-energy waves (see *e.g.* Joarder, Nakariakov, and Roberts, 1997, Terra-Homem, Erdélyi, and Ballai, 2003).

The article is set out as follows. In Section 2 the background equilibrium is outlined, along with the general governing equations and the more simplified governing equations for each layer in the model. The dispersion relation for this model is also derived by implementing the relevant boundary conditions. The incompressible and small wavelength limit are noted here. In Section 3 the cutoff curves defining the domains (*i.e.* windows) of propagation are introduced and a subsequent numerical analysis of the dispersion relation is performed. In Section 4 we conclude.

## Dispersion Relation

### Governing Equation

Consider a plane-parallel background plasma stratified by gravity. Embedded within this plasma is a magnetic field perpendicular to the axis of stratification, *i.e.* the $z$-axis. The plasma has kinetic pressure $p(z)$, density $\rho(z)$, and a steady horizontal flow along the $x$-axis ${\boldsymbol{u}}(z) = (u(z),0,0)$. The gravitational force points in the negative $z$-direction, ${\boldsymbol{g}} = (0,0,-g)$. The magnetic field points in the positive $x$-direction and can vary arbitrarily in the $z$-direction, ${\boldsymbol{B}}(z)=(B(z),0,0)$. The magnetohydrostatic balance is achieved by satisfying the condition

1 $$ \frac{\mathrm{d}}{\mathrm{d}z} \biggl( p(z)+\frac{B^{2}(z)}{2\mu _{0}} \biggr) =-g \rho(z). $$

In a compressible ideal plasma, two-dimensional, linear, isentropic disturbances about the equilibrium take the form

2 $$ {\boldsymbol{\xi}}(x,z,t)=\bigl(\,\widehat{\xi}_{x}(z),0,\widehat{\xi} _{z}(z)\bigr)\mathrm{e}^{i(k_{x}x-\omega t)} $$

for frequency $\omega$ and horizontal wave-number $k_{x}$ and satisfy the second-order ordinary differential equation

3 $$\begin{aligned} &\frac{\mathrm{d}}{\mathrm{d}z} \biggl[ \frac{\rho(z)(c_{\mathrm{s}} ^{2}(z)+v_{\mathrm{A}}^{2}(z))(\Omega^{2}(z)-c_{\mathrm{T}}^{2}(z)k _{x}^{2})}{\Omega^{2}(z)-c_{\mathrm{s}}^{2}(z)k_{x}^{2}} \frac{\mathrm{d}\widehat{\xi}_{z}(z)}{\mathrm{d}z} \biggr] \\ &\quad {}+ \biggl\lceil \rho(z) \bigl(\Omega^{2}(z)-v_{\mathrm{A}}^{2}(z)k_{x}^{2}\bigr) \\ & \quad{} -\frac{g^{2}k_{x}^{2}\rho(z)}{\Omega^{2}(z)-c_{\mathrm{s}}^{2}(z)k _{x}^{2}}-\frac{\mathrm{d}}{\mathrm{d}z} \biggl( \frac{gk_{x}^{2}\rho(z)c _{\mathrm{s}}^{2}(z)}{\Omega^{2}(z)-c_{\mathrm{s}}^{2}(z)k_{x}^{2}}\biggr) \biggr\rfloor \widehat{\xi}_{z}(z)=0, \end{aligned}$$

where $\gamma=5/3$ is the adiabatic index, and $\mu_{0}$ is the magnetic permeability of free-space. Here,

4 $$ c_{\mathrm{s}}(z)= \biggl( \frac{\gamma p(z)}{\rho(z)} \biggr) ^{1/2} $$

is the sound speed,

5 $$ v_{\mathrm{A}}(z)=\frac{B(z)}{(\mu_{0}\rho(z))^{1/2}} $$

is the Alfvén speed,

6 $$ c_{\mathrm{T}}(z)=\frac{c_{\mathrm{s}}(z)v_{\mathrm{A}}(z)}{(c_{ \mathrm{s}}^{2}(z)+v_{\mathrm{A}}(z)^{2})^{1/2}} $$

is the tube speed, and

7 $$ \Omega(z)=\omega-u(z)k_{x} $$

is the Doppler-shifted wave frequency.

### Equilibrium

We assume a single magnetic interface located at $z=0$. The upper isothermal (with temperature $T_{0}$) region ($z>0$) is permeated by an exponentially decreasing horizontal magnetic field $B_{0}(z)$ with the assumption that the Alfvén speed ($v_{\mathrm{A}}$) is constant. The field-free lower region ($z<0$) is also isothermal (but of a different temperature, $T_{\mathrm{e}}$) with a parallel constant steady flow $u_{\mathrm{e}}$.

**Figure 1:**
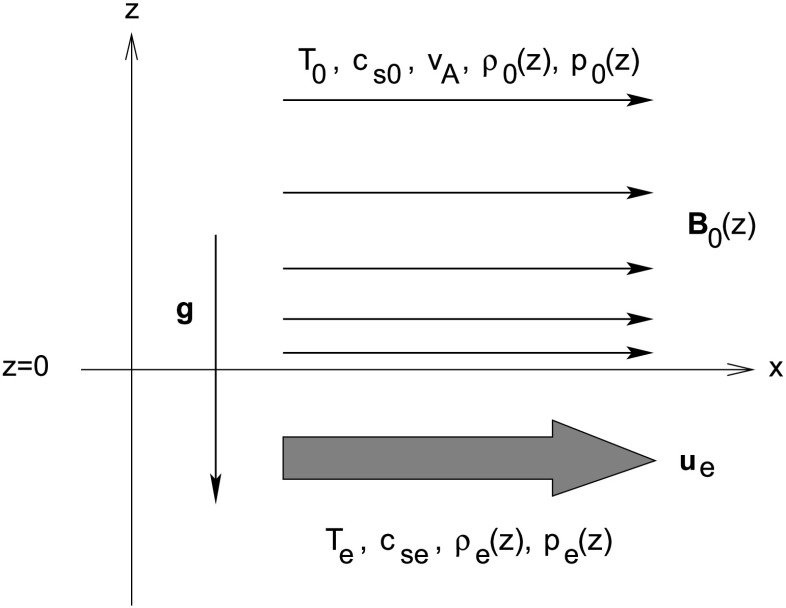
Equilibrium model of a single magnetic interface at $z=0$ in a gravitationally stratified atmosphere, with an exponentially decreasing horizontal magnetic field, $B_{0}(z)$, in $z>0$ and a horizontal constant equilibrium flow, $u_{\mathrm{e}}$, in $z<0$. The temperatures $T_{0}$ and $T_{\mathrm{e}}$ either side of the interface are approximated as isothermal.

Quantities above the interface (in $z>0$) are denoted by the subscript ‘0’, and quantities below (in $z<0$) by the subscript ‘e’ (see Figure 1):

8 $$ T(z), c_{\mathrm{s}}(z), v_{\mathrm{A}}(z), u(z) = \left \{ \textstyle\begin{array}{l@{\quad}l} T_{0}, c_{\mathrm{s}0}, v_{\mathrm{A}}, 0, & z>0 , \\ T_{\mathrm{e}}, c_{\mathrm{se}}, 0, u_{\mathrm{e}}, & z< 0 , \end{array}\displaystyle \right . $$

where $T_{0}$, $T_{\mathrm{e}}$, $c_{\mathrm{s}0}$, $c_{\mathrm{se}}$, $v_{\mathrm{A}}$, and $u_{\mathrm{e}}$ are all constants. Here, ${c_{\mathrm{s}0} = (\gamma p_{0}/\rho_{0})^{1/2}}$ and $v_{\mathrm{A}} = B_{0}/ (\mu_{0} \rho_{0})^{1/2}$ are the sound and Alfvén speeds in the magnetic atmosphere, respectively, and $c_{\mathrm{se}}= (\gamma p_{\mathrm{e}}/\rho_{\mathrm{e}})^{1/2}$ is the sound speed in the field-free region. The following notations are made: $\rho_{0}=\rho_{0}(0_{+})$, $\rho_{\mathrm{e}}=\rho_{\mathrm{e}}(0_{-})$, $p_{0}=p_{0}(0_{+})$, $p_{\mathrm{e}}=p_{\mathrm{e}}(0_{-})$, and $B_{0}=B_{0}(0_{+})$.

The ideal gas law (supplemented by Equation (1) showing that the background varies in the $z$-direction) gives the relationship between the equilibrium pressure, density, and temperature:

9 $$ p(z) = \frac{k_{\mathrm{B}}}{m_{\mathrm{av}}} \rho(z) T(z), $$

where $k_{\mathrm{B}}$ is Boltzmann’s constant, and $m_{\mathrm{av}}$ the mean particle mass of the plasma. This equation, coupled with the conditions that the sound and Alfvén speeds are constants and that both plasmas are isothermal, yields density and magnetic field profiles with forms

10 $$ \rho(z), B(z) = \left \{ \textstyle\begin{array}{l@{\quad}l} \rho_{0} \mathrm{e}^{-z/H_{B}}, B_{0} \mathrm{e}^{-z/2 H_{B}}, & z>0 , \\ \rho_{\mathrm{e}} \mathrm{e}^{-z/H_{\mathrm{e}}}, 0, & z< 0 . \end{array}\displaystyle \right . $$

Here, ${H_{B}=c_{\mathrm{s}0}^{2}/\Gamma g}$ and ${H_{\mathrm{e}}=c _{\mathrm{se}}^{2}/\gamma g}$ are the isothermal density scale-heights above and below the interface, respectively, and

11 $$ \Gamma=\frac{2 \gamma\beta}{\gamma+ 2\beta} $$

is the magnetically modified adiabatic exponent with ${\beta=c_{ \mathrm{s}0}^{2}/v_{\mathrm{A}}^{2}}$. We note that in the limit of zero magnetic field $\Gamma=\gamma$ and $H_{B}=H_{0}=c_{\mathrm{s}0}^{2}/\gamma g$.

### Region with Magnetic Field

Applying Equation (3) to the region with magnetic field ($z>0$) results in the second-order differential equation

12 $$ \frac{\mathrm{d}^{2} \widehat{\xi}_{z}(z)}{\mathrm{d} z^{2}} - \frac{1}{H _{B}} \frac{\mathrm{d} \widehat{\xi}_{z}(z)}{\mathrm{d}z} + A_{B} \widehat{\xi}_{z}(z) = 0, \quad z>0, $$

where

13 $$ A_{B} = \frac{(\Gamma-1) g^{2} k_{x}^{2} +(\omega^{2}-k_{x}^{2} c _{\mathrm{s}0}^{2})(\omega^{2}-k_{x}^{2} v_{\mathrm{A}}^{2})}{(c_{ \mathrm{s}0}^{2}+v_{\mathrm{A}}^{2})(\omega^{2}-k_{x}^{2} c_{ \mathrm{T}}^{2})}. $$

Equation (12) has constant coefficients and possesses the general solution

14 $$ \widehat{\xi}_{z}(z) = d_{1} \,\mathrm{exp}\biggl( \frac{1}{2H_{B}} + M_{0} \biggr) z + d_{2} \,\mathrm{exp} \biggl( \frac{1}{2H_{B}} - M_{0} \biggr) z, \quad z>0, $$

where

15 $$ M_{0} = \frac{ \sqrt{1-4 A_{B} H_{B}^{2}} }{2 H_{B}}, $$

and $d_{1}$ and $d_{2}$ are arbitrary constants.

### Non-Magnetic Region with Background Flow

In the field-free region with the equilibrium bulk motion ($z<0$), Equation (3) reduces to

16 $$ \frac{\mathrm{d}^{2} \widehat{\xi}_{z}(z)}{\mathrm{d} z^{2}} - \frac{1}{H _{\mathrm{e}}} \frac{\mathrm{d} \widehat{\xi}_{z}(z)}{\mathrm{d}z} + A_{\mathrm{e}} \widehat{\xi}_{z}(z) = 0, \quad z< 0, $$

where

17 $$ A_{\mathrm{e}} = \frac{(\gamma-1) g^{2} k_{x}^{2} + \Omega^{2} ( \Omega^{2}-k_{x}^{2} c_{\mathrm{se}}^{2})}{\Omega^{2} c_{\mathrm{se}} ^{2}}, $$

with

18 $$ \Omega= \omega- k_{x} u_{\mathrm{e}}. $$

Equation (16) possesses the general solution

19 $$ \widehat{\xi}_{z}(z) = d_{3} \exp \biggl( \frac{1}{2H_{\mathrm{e}}} + M_{\mathrm{e}} \biggr) z + d_{4} \exp \biggl( \frac{1}{2H_{\mathrm{e}}} - M_{\mathrm{e}} \biggr) z, \quad z< 0, $$

where

20 $$ M_{\mathrm{e}} = \frac{ \sqrt{1-4 A_{\mathrm{e}} H_{\mathrm{e}}^{2}} }{2 H_{\mathrm{e}}}, $$

and $d_{3}$ and $d_{4}$ are arbitrary constants.

### General Dispersion Relation

We require that the total kinetic [$\rho(z) \hat{u}_{1z}^{2}(z)$] plus magnetic [$B_{0}(z)B_{0}'(z) \hat{u}_{1z}(z) + B_{0}^{2}(z){u}_{1z}'(z)$] energy density remains finite as $|z| \rightarrow \infty$, while we assume that ${4 A_{B} H_{B}^{2} < 1}$ and ${4 A_{\mathrm{e}} H_{\mathrm{e}}^{2} < 1}$ (see Section 3.1 for a discussion on these latter conditions). Here,

21 $$ \widehat{u}_{1z}(z)=\frac{i\widehat{\xi}_{z}(z)}{\Omega(z)}. $$

These conditions imply that $d_{1}=d_{4}=0$. Thus, the amplitude of the vertical Lagrangian displacement component, $\widehat{\xi}_{z}(z)$, in the two regions is given by

22 $$ \widehat{\xi}_{z}(z) = \left \{ \textstyle\begin{array}{l@{\quad}l} d_{2} \exp ( \frac{1}{2H_{B}} - M_{0} ) z, & z>0 , \\ d_{3} \exp ( \frac{1}{2H_{\mathrm{e}}} + M_{\mathrm{e}} ) z, & z< 0 . \end{array}\displaystyle \right . $$

Equation (22) implies that $\widehat{\xi}_{z}(z) \rightarrow 0$ as $z \rightarrow-\infty$, i.e. $\widehat{u}_{1z}(z)$ is exponentially diminishing for $z<0$. However, for $z>0$ $\widehat{\xi}_{z}(z)$ is exponentially decreasing only if $A_{B}<0$ (see Equation (15)), elsewhere it is exponentially growing with height.

The boundary conditions to be applied are the continuity of the vertical component of the Lagrangian displacement and the continuity of the Lagrangian perturbation of total (gas plus magnetic) pressure across the interface at $z=0$:

23 $$\begin{aligned} \bigl\{ \widehat{\xi}_{z}(z) \bigr\} _{z=0} = & 0 , \end{aligned}$$

24 $$\begin{aligned} \bigl\{ \widehat{p}_{\mathrm{T1}}(z) - \rho(z) g \widehat{\xi}_{z}(z) \bigr\} _{z=0} = & 0 , \end{aligned}$$

where

25 $$\begin{aligned} \widehat{p}_{\mathrm{T1}}(z) =-\rho(z) \frac{(c_{\mathrm{s}} ^{2}(z) + v_{\mathrm{A}}^{2}(z)) (\Omega^{2}(z) - k_{x}^{2} c_{ \mathrm{T}}^{2}(z))}{\Omega^{2}(z) - k_{x}^{2} c_{\mathrm {s}}^{2}(z)}\frac{ \mathrm{d}\widehat{\xi}_{z}(z)}{\mathrm{d}z} +\frac{ \Omega^{2}(z) g \rho(z)}{\Omega^{2}(z) - k_{x}^{2} c_{ \mathrm{s}}^{2}(z)} \widehat{\xi}_{z}(z) \end{aligned}$$

is the Eulerian perturbation of total pressure. Applying the two matching conditions, Equations (23) and (24), to solution (22), we obtain the transcendental dispersion relation

26 $$\begin{aligned} &\frac{\rho_{0}(c_{\mathrm{s}0}^{2}+v_{\mathrm{A}}^{2})(\omega^{2}-c _{\mathrm{T}}^{2}k_{x}^{2})}{\omega^{2}-c_{\mathrm{s}0}^{2}k_{x}^{2}} \biggl( M_{0}- \frac{1}{2H_{B}} \biggr) +\frac{\rho_{0}gk_{x}^{2}c_{ \mathrm{s}0}^{2}}{\omega^{2}-c_{\mathrm{s}0}^{2}k_{x}^{2}} \\ &\quad =\frac{\rho_{\mathrm{e}} c_{\mathrm{se}}^{2}}{\Omega^{2}-c^{2}_{ \mathrm{se}}k_{x}^{2}} \biggl[ gk_{x}^{2}- \biggl( \frac{1}{2H_{\mathrm{e}}}+M _{\mathrm{e}} \biggr) \Omega^{2} \biggr] , \end{aligned}$$

where $c_{\mathrm{T}} = c_{\mathrm{s0}} v_{\mathrm{A}} / (c_{ \mathrm{s}0}^{2}+v_{\mathrm{A}}^{2})^{1/2}$ is the tube speed in the magnetic atmosphere. Equation (26) describes the parallel propagation of surface waves at a single magnetic interface in a gravitationally stratified atmosphere under the assumption of constant Alfvén speed in the upper isothermal magnetic region and a constant flow in the field-free lower isothermal region.

### Incompressible Limit

Equation (26) can be written in a more useful and familiar form:

27 $$\begin{aligned} \frac{\omega^{2}}{k_{x}^{2}} =& \dfrac{\rho_{0}}{\rho_{0} + \rho_{\mathrm{e}}\frac{ ( M_{\mathrm{e}} + 1/2H_{\mathrm{e}} ) m_{0}}{ ( M_{0} - 1/2H_{B} )m_{\mathrm{e}}}}v_{\mathrm{A}}^{2} -g \dfrac{\frac{\rho_{0}c_{\mathrm{s}0}^{2}}{ ( \omega^{2}-c_{\mathrm{s0}}^{2}k_{x}^{2} ) } - \frac{\rho_{\mathrm {e}}c_{\mathrm{se}}^{2}}{ ( \Omega^{2}-c_{\mathrm{se}}^{2}k_{x}^{2} ) }}{\rho_{0}\frac{ ( M_{0}-1/2H_{B} ) }{m_{0}} +\rho_{\mathrm{e}}\frac{ ( M_{\mathrm{e}}+1/2H_{\mathrm{e}} ) }{m_{\mathrm{e}}}}, \end{aligned}$$

where

28 $$ m_{0}= \dfrac{ ( \omega^{2}-v_{\mathrm{A}}^{2}k_{x}^{2} ) ( \omega^{2}-c_{\mathrm{s0}}^{2}k_{x}^{2} ) }{ ( c_{\mathrm {s0}}^{2}+v_{\mathrm{A}}^{2} ) ( \omega^{2}-c_{\mathrm {T}}^{2}k_{x}^{2} ) }, $$

and

29 $$ m_{\mathrm{e}}=\frac{ ( \Omega^{2}-c_{\mathrm {se}}^{2}k_{x}^{2} ) \omega^{2}}{c_{\mathrm{se}}^{2}\Omega^{2}}. $$

In the incompressible limit, the sound speeds in both layers tend towards infinity, *i.e.* $c_{\mathrm{s0}},c_{ \mathrm{se}}\to\infty$. Therefore $m_{\mathrm{e}}\to-k_{x}^{2} \omega^{2}/\Omega^{2}$, $m_{0}\to-k_{x}^{2}$ and $M_{\mathrm{e}},M _{0}\to k_{x}$. Taking the special case of uniform distributions of density, such that $\rho_{0}(z)=\rho_{0}$ and $\rho_{\mathrm{e}}(z)= \rho_{\mathrm{e}}$ as in Sengottuvel and Somasundaram (2001), Equation (27) reduces to the second-order polynomial for the horizontal phase speed ($\omega/k_{x}$):

30 $$ \biggl( \dfrac{\omega}{k_{x}} \biggr) ^{2}-\frac{2u_{\mathrm{e}}}{1+ \rho_{\mathrm{r}}} \dfrac{\omega}{k_{x}}+ \biggl( \frac{u_{\mathrm{e}} ^{2}}{1+\rho_{\mathrm{r}}}-\frac{v_{\mathrm{A}}^{2}\rho_{\mathrm{r}}}{1+ \rho_{\mathrm{r}}}+ \frac{g}{k_{x}} \biggl( \frac{1-\rho_{\mathrm{r}}}{1+ \rho_{\mathrm{r}}} \biggr) \biggr) =0. $$

Here, $\rho_{\mathrm{r}}=\rho_{0}/\rho_{\mathrm{e}}$. When we solve Equation (30), the solution for the phase speed can be written as

31 $$ \frac{\omega}{k_{x}}=\frac{u_{\mathrm{e}}}{1+\rho_{\mathrm{r}}} \biggl[ 1 \pm \biggl\{ \rho_{\mathrm{r}} \biggl( \frac{(1+\rho_{\mathrm{r}}) ( v_{\mathrm{A}}^{2}\rho_{\mathrm{r}}+\frac{g}{k_{x}}(1-\rho_{\mathrm {r}}) ) }{\rho_{\mathrm{r}}u_{\mathrm{e}}^{2}}-1 \biggr) \biggr\} ^{1/2} \biggr] . $$

This dispersion relation agrees well with that derived in Sengottuvel and Somasundaram (2001), except that their flow is in the upper layer. A simple Galilean transformation shows a full algebraic agreement. It is immediately evident from Equation (31) that when the term inside the square root becomes lower than zero, instability occurs. This can be written in the form of an inequality,

32 $$ ( 1+\rho_{\mathrm{r}} ) \biggl( v_{\mathrm{A}}^{2}+ \frac{g}{k _{x}}\frac{(1-\rho_{\mathrm{r}})}{\rho_{\mathrm{r}}} \biggr) < u_{ \mathrm{e}}^{2}. $$

The critical wave number (denoted $k_{x,\mathrm{c}}$) for the Kelvin–Helmholtz instability is then given by

33 $$ {k_{x,\mathrm{c}}}= \dfrac{(1-\rho_{\mathrm{r}})g}{\rho_{\mathrm{r}} ( u_{\mathrm {e}}^{2}-v_{\mathrm{A}}^{2}\rho_{\mathrm{r}} ) }. $$

Clearly, if the plasma in the top layer is lighter than the plasma in the bottom layer, then $u_{\mathrm{e}}^{2}>v_{\mathrm{A}}^{2} \rho_{\mathrm{r}}$ such that ${k_{x,\mathrm{c}}}$ is positive. The associated critical phase speed (denoted $v_{\mathrm{ph,c}}$) at which the Kelvin–Helmholtz instability occurs is given by

34 $$ v_{\mathrm{{ph},c}}=\frac{u_{\mathrm{e}}}{1+\rho_{\mathrm{r}}}. $$

### Small Wavelength ($k_{x}H_{\mathrm{e}}\to\infty$) and Cold Plasma ($\beta=0$) Limit

If the limit as $k_{x}H_{\mathrm{e}}\to\infty$ of Equation (26) is taken, then it reduces to the following equation:

35 $$\begin{aligned} \rho_{\mathrm{r}}^{2}\dfrac{ ( \widehat{\omega}^{2}-\widehat {v}_{\mathrm{A}}^{2} ) ( \widehat{\omega}^{2}-\widehat{c}_{\mathrm{T}}^{2} ) ( \widehat{c}_{\mathrm{s0}}^{2}+\widehat{v}_{\mathrm{A}}^{2} ) }{\widehat{\omega}^{2} -\widehat{c}_{\mathrm{s0}}^{2}}= \dfrac{\widehat{\Omega}^{4}}{\widehat{\Omega}^{2}-1}. \end{aligned}$$

Here we have introduced the following dimensionless quantities, whilst also noting the dimensionless flow speed for later:

36 $$\begin{aligned} \widehat{\omega}=\frac{\omega}{k_{x}c_{\mathrm{se}}}, \quad \widehat{\Omega}=\frac{\Omega}{k_{x}c_{\mathrm{se}}}, \quad \widehat{v}_{\mathrm{A}}=\frac{v_{\mathrm{A}}}{c_{\mathrm{se}}}, \quad\widehat{c}_{\mathrm{s0}}= \frac{c_{\mathrm{s0}}}{c_{\mathrm{se}}},\quad\widehat{c}_{\mathrm {T}}=\frac{c _{\mathrm{T}}}{c_{\mathrm{se}}}, \quad \widehat{u}_{\mathrm{e}}=\frac{u _{\mathrm{e}}}{c_{\mathrm{se}}}. \end{aligned}$$

We note that Equation (35) is derived by squaring terms so that spurious roots may have developed. This equation is still transcendental, however, therefore the cold plasma (*i.e.* $c_{\mathrm{s0}}=0$) limit is taken for analytic progress. This removes the slow surface mode, but the fast mode is retained. When this limit is applied, Equation (35) results in the following fourth-order polynomial in $\Omega$:

37 $$\begin{aligned} \begin{aligned} &\widehat{\Omega}^{4} \biggl( 1- \frac{2\rho_{\mathrm{r}}}{\gamma} \biggr) -\frac{4\rho_{\mathrm{r}}\widehat{u}_{\mathrm{e}}}{\gamma} \widehat{\Omega}^{3}- \widehat{\Omega}^{2} \biggl( \frac{2\rho_{ \mathrm{r}}}{\gamma}\widehat{u}_{\mathrm{e}}^{2}- \frac{4}{\gamma^{2}}-\frac{2\rho_{\mathrm{r}}}{\gamma} \biggr) +\frac{4\rho_{\mathrm{r}}\widehat{u}_{\mathrm{e}}}{\gamma} \widehat{\Omega} + \biggl( \frac{2\rho_{\mathrm{r}}\widehat{u}_{ \mathrm{e}}^{2}}{\gamma}- \frac{4}{\gamma^{2}} \biggr) =0. \end{aligned} \end{aligned}$$

Pressure balance in this limit gives $\widehat{v}_{\mathrm{A}}^{2}=2/ \rho_{\mathrm{r}}\gamma$. Again, Equation (37) is still too difficult to solve analytically. Two separate approximations to Equation (37) are then taken for analytic progress:

1. The limit of small flow *i.e.* $\widehat{u}_{\mathrm {e}}=\epsilon $, where $\epsilon\ll1$.
2. A large discontinuity in density *i.e.* $\rho_{\mathrm{r}}= \epsilon$, where $\epsilon\ll1$.

#### Limit of Small Flow

In the limit of small flow, the frequency is approximated as

38 $$\begin{aligned} \widehat{\omega}=\pm\widehat{\Omega}_{0}+\hat{u}_{\mathrm{e}} \dfrac {\widehat{\Omega}_{0}^{2} ( 2-\rho_{\mathrm{r}}^{2}\widehat{v}_{\mathrm{A}}^{2} ) +\rho _{\mathrm{r}}^{2}\widehat{v}_{\mathrm{A}}^{2}}{\rho_{\mathrm {r}}\widehat{v}_{\mathrm{A}}^{2} ( \rho_{\mathrm{r}}^{2}(1-\widehat {v}_{\mathrm{A}}^{2})^{2}+4 ) ^{1/2}}+ \mathrm{O}\bigl(\widehat{u}_{\mathrm{e}}^{2}\bigr), \end{aligned}$$

where

39 $$\begin{aligned} \widehat{\Omega}_{0}^{2}=- \dfrac{\rho_{\mathrm{r}}^{2}\widehat{v}_{\mathrm{A}}^{2} ( 1+\widehat{v}_{\mathrm{A}}^{2} ) }{2 ( 1-\rho_{\mathrm {r}}^{2}\widehat{v}_{\mathrm{A}}^{2} ) } \biggl( 1- \biggl( 1+ \dfrac{4 ( 1-\rho_{\mathrm{r}}^{2}\widehat{v}_{\mathrm {A}}^{2} ) }{\rho_{\mathrm{r}}^{2} ( 1+\widehat{v}_{\mathrm {A}}^{2} ) ^{2}} \biggr) ^{1/2} \biggr) . \end{aligned}$$

Equation (38) must satisfy the following conditions for surface waves to exist:

$$ (1)\quad\widehat{\omega}^{2}< \widehat{v}_{\mathrm{A}}^{2}, \qquad (2) \quad\widehat{\Omega}^{2}< 1. $$

As there is gravity in the system, the density ratio can only range between the values of zero and one, *i.e.* $0\leq\rho_{ \mathrm{r}}\leq1$. We therefore have a minimum value of $\widehat{v} _{\mathrm{A}}^{2}=2/\gamma$. This is greater than one (taking the value of the adiabatic index, $\gamma$, to be $5/3$), and therefore we only need to fulfil condition (2) above. We find that solutions do not exist for $\widehat{\Omega}_{0}^{2}=1$ and that the function $\widehat{\Omega}_{0}^{2}(\rho_{\mathrm{r}})$, from Equation (39), is continuous. If a value for $\widehat{\Omega}_{0}^{2}$ can be found that is lower than one, then all values of $\widehat{\Omega}_{0}^{2}$ must be lower than one. In the limit $\rho_{\mathrm{r}}\to0$,

40 $$\begin{aligned} \widehat{\Omega}_{0}^{2} =& - \frac{2}{\gamma^{2}} \bigl( 1-\bigl(1+\gamma ^{2}\bigr)^{1/2}\bigr) \\ \approx& 0.68. \end{aligned}$$

**Figure 2:**
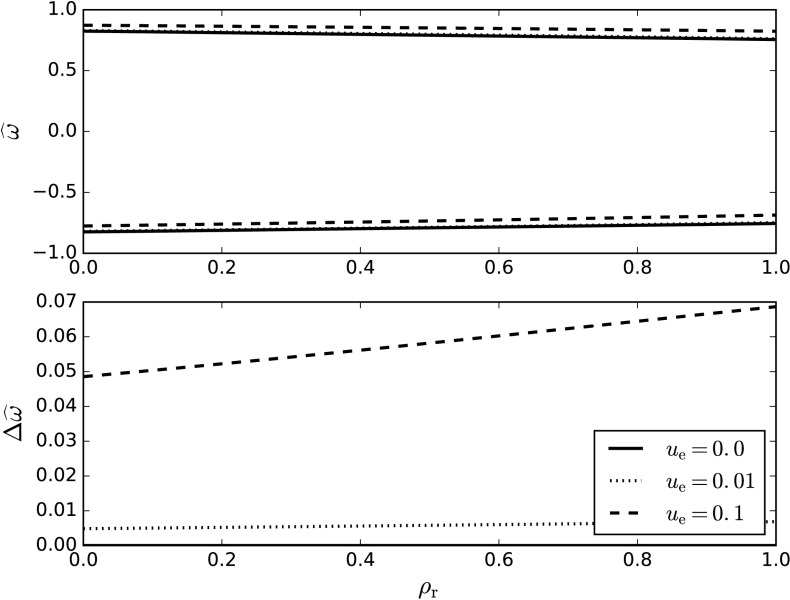
The upper panel shows the frequency computed from Equation (38). The lower panel shows the corresponding frequency shift ($\Delta\widehat{\omega}$) that is due to the small flow contribution. The legend indicates the lines that correspond to the values $u_{\mathrm{e}}=0.0,0.01,\text{and }0.1$. The adiabatic index, $\gamma$, is taken to be $5/3$.

Surface waves exist in this limit, and therefore surface waves must exist everywhere for $\widehat{u}_{\mathrm{e}}= 0$. The upper part of Figure 2 plots the frequencies calculated from Equation (38) against the ratio of the density in the upper layer to the density in the lower layer for three values of the flow, $\widehat{u}_{\mathrm{e}}=0.0, 0.01, \mbox{and } 0.1$. The lower part of the figure shows the corresponding frequency shift for both the backward- and forward-propagating waves. The frequencies clearly do not rise above the value of 1.0 and therefore satisfy the surface waves condition. The forward-propagating wave is accelerated in its direction of propagation, whereas the backward wave is decelerated.

#### Limit of Low Density Ratio

In the limit of a low density ratio between the layers, the frequency can be approximated by the following equation:

41 $$\begin{aligned} \widehat{\omega}= ( \pm\widehat{\Omega}_{0}+\widehat{u}_{ \mathrm{e}} ) \biggl( 1\pm\rho_{\mathrm{r}} \dfrac{\gamma ( \widehat{\Omega}_{0}^{2}-1 ) ( \pm\hat {\Omega}_{0}+\widehat{u}_{\mathrm{e}} ) }{4\widehat{\Omega }_{0} ( \pm ( 1+\gamma^{2} ) ^{1/2} ) } \biggr) . \end{aligned}$$

Here,

42 $$\begin{aligned} \widehat{\Omega}_{0}= \biggl( \dfrac{2 ( -1\pm ( 1+\gamma ^{2} ) ^{1/2} ) }{\gamma^{2}} \biggr) ^{1/2}. \end{aligned}$$

Equation (42) is the Doppler-shifted frequency, which means that it therefore is the frequency when there is no flow present. This equation agrees with the equation given in Roberts (1981b), which was derived for surface waves at a magnetic interface with the same structure as in this article, but with neither flow nor stratification (the $k_{x}H_{\mathrm{e}}\to\infty$ limit is equivalent to the zero-gravity limit). The approximations taken in that article were $v_{\mathrm{A}}\gg c_{\mathrm{s0}}$, $c_{\mathrm{se}}$. This is equivalent to the cold plasma approximation with a low density ratio taken here.

**Figure 3:**
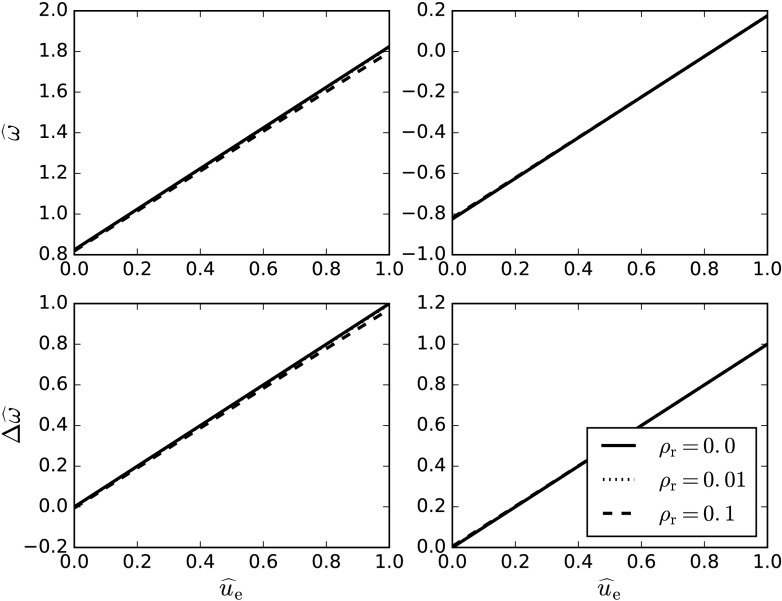
The upper left panel shows the frequency plotted against dimensionless flow speed, $\widehat{u}_{\mathrm{e}}$, for the forward-propagating solution. The upper right panel is the same, but for the backward-propagating solution. The two panels at the bottom depict the frequency shift for the forward- and backward-propagating solutions on the left and right, respectively. Each diagram has been plotted for separate values of the density ratio, $\rho_{\mathrm{r}}$. The adiabatic index, $\gamma$, is taken to be $5/3$.

Again, the frequency satisfies the condition of surface waves to zeroth order. Figure 3 shows the frequencies given by Equation (41) for both the forward- and backward-propagating solutions, varying with dimensionless flow speed, $\widehat{u}_{\mathrm{e}}$. Figure 3 also depicts the corresponding frequency shift associated with the changing flow.

The frequency for both the backward and forward waves increases linearly with the flow, as does the frequency shift. When the density ratio is increased, the frequency shift for the backward-propagating wave is largely unaffected. However, for the forward-propagating wave, the frequency shift is slightly lowered. For a high enough flow speed the backward-propagating wave can change its direction of propagation, as shown by Figure 3, when it takes a positive value of $\widehat{\omega}$.

## Numerical Solution of the Dispersion Relation

### Cut-off Curves

The dispersion relation, Equation (26), is solved subject to the constraints that $\omega\ne\pm k_{x} c_{\mathrm{s0}}$, $\omega\ne k_{x}(u_{\mathrm{e}} \pm c_{\mathrm{se}})$ and that

43 $$ 1 - 4 A_{B} H_{B}^{2} > 0 \quad \mbox{and} \quad 1 - 4 A_{\mathrm{e}} H_{\mathrm{e}}^{2} > 0. $$

These two latter constraints are required for surface wave solutions, *i.e.* evanescent (non-propagating) modes in the $z$ direction. This means that in the $\omega\mbox{--}k_{x}$ plane these constraints (where both are fulfilled) determine those permitted regions of the $(\omega,k_{x})$ parameter space of propagation where magnetoacoustic-gravity surface modes given by Equation (26) may propagate. The boundary curves to these regions represent cut-off curves (cut-off frequencies) for the modes. Outside these regions, where either $(1 - 4 A_{B} H_{B}^{2})$ or $(1 - 4 A_{\mathrm{e}} H_{ \mathrm{e}}^{2})$ is negative, spatially oscillating leaky-modes arise in which we are not interested.

The condition $1 - 4 A_{\mathrm{e}} H_{\mathrm{e}}^{2} > 0$ gives rise to the cut-off curves $R_{1}$, $R_{2}$, $R_{3}$, and $R_{4}$ given by

44 $$ R_{1,2} = u_{\mathrm{e}}/c_{\mathrm{se}}+P_{1,2} , \quad \quad R_{3,4} = u_{\mathrm{e}}/c_{\mathrm{se}}-P_{2,1} , $$

where

45 $$ P_{1,2}^{2} = \frac{1 + 4 k_{x}^{2} H_{\mathrm{e}}^{2} \mp \sqrt{(1 + 4 k_{x}^{2} H_{\mathrm{e}}^{2})^{2} - 64 \frac{\gamma-1}{\gamma^{2}}k_{x}^{2} H_{\mathrm{e}}^{2}} }{8 k_{x} ^{2} H_{\mathrm{e}}^{2}}. $$

We note that $P_{2}>P_{1}>0$. The regions where $1 - 4 A_{\mathrm{e}} H_{\mathrm{e}}^{2} > 0$ is satisfied are defined by either

46 $$ R_{1} < \frac{\omega}{k_{x} c_{\mathrm{se}}} < R_{2} $$

or

47 $$ R_{3} < \frac{\omega}{k_{x} c_{\mathrm{se}}} < R_{4}. $$

The other condition, $1 - 4 A_{B} H_{B}^{2} > 0$, generates the cut-off curves $R_{5} \text{ and} R_{6}$, $\text{which}$ satisfy the equation

48 $$ \frac{ 4 \frac{c_{\mathrm{s0}}^{4}}{c_{\mathrm{se}}^{4}} }{\Gamma^{2}} \biggl[ \frac{ \Gamma-1 + \gamma^{2} k_{x}^{2} H_{\mathrm{e}}^{2} ( R_{5,6}^{2}-\frac{c_{\mathrm{s0}}^{2}}{c_{\mathrm{se}}^{2}} ) ( R_{5,6}^{2}-\frac{v_{\mathrm{A}}^{2}}{c_{\mathrm{se}}^{2}} ) }{ ( \frac{c_{\mathrm{s0}}^{2}}{c_{\mathrm{se}}^{2}} + \frac{v _{\mathrm{A}}^{2}}{c_{\mathrm{se}}^{2}} ) ( R_{5,6}^{2} - \frac{c _{\mathrm{T}}^{2}}{c_{\mathrm{se}}^{2}} ) } \biggr] = 1, $$

(note that $R_{6}>R_{5}>0$), and the regions where $1 - 4 A_{B} H_{B} ^{2} > 0$ is met are either

49 $$\begin{aligned} &R_{6} < \frac{\omega}{k_{x} c_{\mathrm{se}}} < \frac{c_{\mathrm{T}}}{c _{\mathrm{se}}}, \end{aligned}$$

50 $$\begin{aligned} &-\frac{c_{\mathrm{T}}}{c_{\mathrm{se}}} < \frac{\omega}{k_{x} c_{ \mathrm{se}}} < -R_{6}, \end{aligned}$$

51 $$\begin{aligned} &\text{max} \biggl( -\frac{c_{\mathrm{T}}}{c_{\mathrm{se}}}, -R_{5} \biggr) < \frac{\omega}{k_{x} c_{\mathrm{se}}} < \text{min} \biggl( \frac{c _{\mathrm{T}}}{c_{\mathrm{se}}}, R_{5} \biggr) , \end{aligned}$$

52 $$\begin{aligned} &\text{max} \biggl( \frac{c_{\mathrm{T}}}{c_{\mathrm{se}}}, R_{5} \biggr) < \frac{\omega}{k_{x} c_{\mathrm{se}}} < R_{6}, \end{aligned}$$

or

53 $$ -R_{6} < \frac{\omega}{k_{x} c_{\mathrm{se}}} < \text{min} \biggl( -\frac{c _{\mathrm{T}}}{c_{\mathrm{se}}}, -R_{5} \biggr) . $$

We note that both positive and negative phase speed ($\omega/k_{x}$) solutions of the dispersion relation are allowed. Now, considering the phase speed regions determined by Equations (46) – (47) and (49) – (53), we can easily deduce the permitted regions where the two constraints written in Equation (43) are fulfilled.

In the limit $k_{x} H_{\mathrm{e}} \rightarrow\infty$ (which is equivalent to $g \rightarrow0$), we obtain

54 $$\begin{aligned} &R_{1} \rightarrow\frac{u_{\mathrm{e}}}{c_{\mathrm{se}}}, \quad \quad R_{2}\rightarrow\frac{u_{\mathrm{e}}}{c_{\mathrm{se}}}+1, \end{aligned}$$

55 $$\begin{aligned} &R_{3} \rightarrow\frac{u_{\mathrm{e}}}{c_{\mathrm{se}}}-1, \quad \quad R_{4} \rightarrow\frac{u_{\mathrm{e}}}{c_{\mathrm{se}}}, \end{aligned}$$

56 $$\begin{aligned} &R_{5} \rightarrow\text{min} \biggl( \frac{c_{\mathrm{s0}}}{c_{\mathrm{se}}}, \frac{v_{\mathrm{A}}}{c_{\mathrm{se}}} \biggr) , \quad \quad R_{6} \rightarrow\text{max} \biggl( \frac{c_{\mathrm{s0}}}{c_{ \mathrm{se}}}, \frac{v_{\mathrm{A}}}{c_{\mathrm{se}}} \biggr). \end{aligned}$$

The cut-off curves are illustrated in the next section.

### Numerical Results

We now examine modes resulting from the numerical solution of the dispersion relation, Equation (26). The scope of the current work is to explore the effect of increasing flow magnitude on the modes, ascending from the value of zero flow.

First of all, we recall that in the absence of gravity and flow, a magnetic interface may support one or two surface modes (depending on the relative temperatures of the media either side of the interface and on the strength of the magnetic field). These are the fast and slow magnetoacoustic (MA) surface waves (Roberts, 1981b). Whereas the slow mode is always present, the fast mode only appears when both $v_{\mathrm{A}} > c_{\mathrm{s0}}$ (*i.e.* the magnetic field is strong enough to dominate the nature of the plasma within the magnetic region) and $c_{\mathrm{se}} > c_{\mathrm{s0}}$ (*i.e.* the field-free region is warmer than the magnetic atmosphere). When these conditions are fulfilled, both waves have sub-Alfvénic phase-speeds ($\omega/k_{x}$).

In the presence of gravity, the counterparts of MA modes arise, which are called magnetoacoustic-gravity (MAG) surface modes. These are the fast and slow MA surface modes modified by the gravity. For a detailed investigation see Miles and Roberts (1992). However, when the field-free region is warmer than the magnetic medium ($c_{\mathrm{se}} > c_{\mathrm{s0}}$), there also appears a third mode, the *f*-mode, which is modified by the magnetic field, although for a limited range of the horizontal wavenumber. Miles and Roberts (1992) showed that as the magnetic field strength is increased, the *f*-mode develops into the fast magnetoacoustic-gravity surface mode. Furthermore, when $v_{\mathrm{A}} > c_{\mathrm{s0}}$ is satisfied, this solution merges with the fast surface-wave solution of the non-stratified ($g=0$) medium at very low wavelengths ($k_{x} H_{\mathrm{e}} \rightarrow\infty$). On the other hand, when the magnetic medium is warmer than the field-free region ($c_{\mathrm{s0}} > c_{\mathrm{se}}$), the *f*-mode is replaced by a surface gravity wave.

Consider now the influence of a background equilibrium flow on the modes discussed above. We solve the dispersion relation, Equation (26), numerically and plot the dimensionless longitudinal phase speed, $\omega/ k_{x} c_{\mathrm{se}}$, as a function of the dimensionless horizontal wavenumber, $k_{x} H_{ \mathrm{e}}$. We set $\gamma=5/3$ throughout.

**Figure 4:**
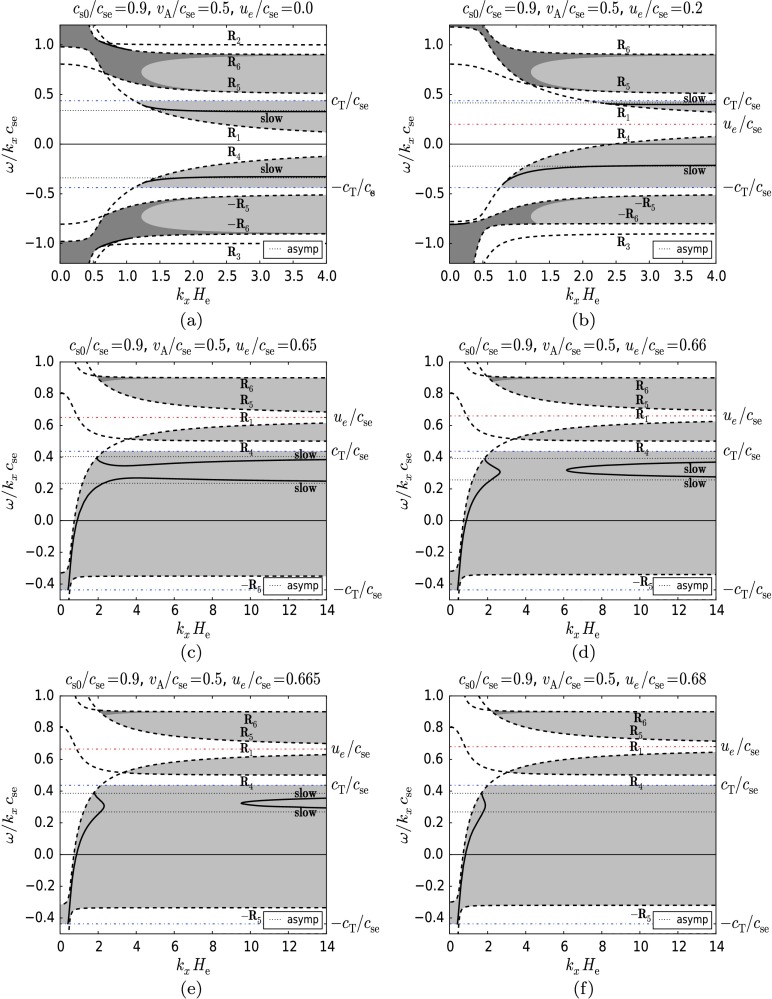
The dimensionless phase speed of MAG surface waves as a function of the dimensionless horizontal wavenumber for $c_{\mathrm{s0}}/c_{\mathrm {se}}=0.9$, $v_{\mathrm{A}}/c_{\mathrm{se}}=0.5$ and (a) $u_{\mathrm{e}}/c_{\mathrm{se}}=0.0$, (b) $u_{\mathrm {e}}/c_{\mathrm{se}}=0.2$, (c) $u_{\mathrm{e}}/c_{\mathrm{se}}=0.65$, (d) $u_{\mathrm{e}}/c_{\mathrm{se}}=0.66$, (e) $u_{\mathrm {e}}/c_{\mathrm{se}}=0.665$, (f) $u_{\mathrm{e}}/c_{\mathrm{se}}=0.68$. The grey regions bounded by the cutoff curves $R_{1}$, $R_{2}$, $R_{3}$, $R_{4}$, $\pm R_{5}$, $\pm R_{6}$, $\pm c_{\mathrm {T}}/c_{\mathrm{se}}$ are regions where surface waves can propagate. Dark grey regions contain modes for which the vertical velocity component is exponentially growing with height in the $z>0$ region. In the light grey regions modes have decreasing vertical velocities with height $z$. The asymptotes to which the modes tend in the short-wavelength limit ($k_{x} H_{\mathrm {e}} \rightarrow\infty$) are shown as well. The effect of increasing flow on the slow magnetoacoustic-gravity mode and the magnetically modified *f*-mode can be seen in panel (a). Note that as the permitted regions of propagation shift upward, the *f*-modes vanish (panels (b), (c)). The forward-propagating slow mode, while keeping its relatively constant position, is transferred to the permitted region of the backward slow mode (panel (c)). The backward-propagating slow mode continues to shift upward (panel (b)) and thus changes its direction of propagation (panel (c)), approaches and finally reaches the upper slow mode and connects with it (panel (d)). The coupled slow modes form two branches, and the gap between them continues to expand with growing flow (panels (d), (e)). After the asymptotes reach each other and cancel out, only the lower branch of the coupled modes remains (panel (f)), which gradually disappears as the flow increases.

Figure 4 shows solutions (thick lines) where $c_{ \mathrm{se}} > c_{\mathrm{s0}}$ (specifically $c_{\mathrm{s0}}/c_{ \mathrm{se}}=0.9$), and $v_{\mathrm{A}} < c_{\mathrm{s0}}$ (specifically $v_{\mathrm{A}}/c_{\mathrm{se}}=0.5$), for six arbitrary but purposefully chosen values of the dimensionless flow, $u_{\mathrm{e}}/c _{\mathrm{se}}$, starting from zero up (Figures 4a – f). These parameters describe a situation at the lower solar atmospheric region where the plasma is slightly hotter in the lower layer. The plasma-beta value is approximately 0.98 and the density ratio ($\rho _{0}/\rho_{\mathrm{e}}$) is 0.98. These parameters can approximate the temperature minimum region in the solar photosphere embedded in an overlying horizontal magnetic field (*e.g.* in the higher parts of the penumbra). The permitted regions of propagation for surface modes (see Section 3.1) bounded by the cut-off curves $R_{1}$, $R_{2}$, $R_{3}$, $R_{4}$, $\pm R_{5}$, and $\pm R_{6}$ (shown as dashed lines) are shaded grey. The dot-dashed line represents the curve $A_{B}=0$ (see Equation (13)) and divides the region of evanescence in the upper medium ($z>0$) into a region (shaded dark grey) where the vertical velocity component is exponentially growing with height, $z$, (here $A_{B}>0$), from a region (shaded light grey) where it decreases with height (here $A_{B}<0$). We note again that in the lower region, $z<0$, the vertical velocity component always becomes smaller with height.

The dot-dashed horizontal lines in Figure 4 correspond to $\omega=\pm k_{x} c_{\mathrm{T}}$ and the dotted horizontal lines correspond to the asymptotes to which the MAG surface modes tend as $k_{x} H_{\mathrm{e}} \rightarrow\infty$. The limit $k_{x} H_{ \mathrm{e}} \rightarrow\infty$ is equivalent to $g \rightarrow0$, where the asymptotes can be determined by solving the non-gravity dispersion relation that is obtained from the dispersion relation (26) in the limit of zero gravity.

We note that with the parameters chosen as $c_{\mathrm{s0}}/c_{ \mathrm{se}}=0.9$ and $v_{\mathrm{A}}/c_{\mathrm{se}}=0.5$, only the slow surface mode can propagate in the zero-gravity limit and therefore for large $k_{x} H_{\mathrm{e}}$. Thus, in Figure 4a (which corresponds to the static background case) we show that the slow MAG mode (the fast mode is absent) is only present within the light grey region ($A_{B}<0$). At lower wavenumbers (for a limited range of wavenumbers) the *f*-mode (modified by the magnetic field) is visible. It lies entirely within the dark grey region ($A_{B}>0$) and therefore has a growing vertical velocity in the upper medium ($z>0$).

Modes with negative phase speeds correspond to waves propagating in the opposite, backwards, direction. Here, in the static case, forward- (positive phase speed) and backward-propagating (negative phase speed) waves are symmetrical counterparts to the $\omega/ (k_{x} c_{ \mathrm{se}})=0$ axis. In the remaining figures, we examine the effect of increasing the equilibrium flow on the symmetrical modes of the static equilibrium.

The cut-off curves $R_{1}$, $R_{2}$, $R_{3}$, $\text{and } R_{4}$ are shifted upwards as the flow increases (see Equation (44)), but the curves $\pm R_{5}$, $\pm R_{6}$ (Equation (48)) and $A_{B}=0$ (Equation (13)) do not change their position. This results in a similar upward shift of the permitted regions of wave propagation as well as in the deformation of their shape. We may observe that permitted regions may disappear together with the modes propagating in them as, for example, in Figures 4b, c where the *f*-modes vanish. New permitted regions may also appear, but it does not necessarily follow that new modes also appear (see Figure 4c, where the permitted regions bounded by the curves $R_{1} \text{ and } R_{6}$ and $R_{5} \text{ and } R_{4}$ appear without new modes).

The flow also modifies the frequency (and the phase speed) of the modes. However, frequency shifts of various modes may differ in magnitude. In the series of panels from Figure 4a – f, the backward-propagating slow mode shifts together with the flow and even changes its direction of propagation, thus becoming a forward-propagating (in the same direction as the flow) mode (see Figure 4c).

On the other hand, the forward-propagating slow mode mainly retains its position (*i.e.* its phase speed), while the permitted regions “pass through” it as the flow changes. The permitted region that originally contains this mode (bounded by the curves $R_{1}$ and $c_{\mathrm{T}}/c_{\mathrm{se}}$) gradually disappears as the flow increases (see Figures 4a, b), and in the end, the mode is transferred to the permitted region containing the other slow mode “travelling upward” with the flow (Figure 4c).

In what follows, the slow mode with lower phase speed continuously approaches the upper slow mode as the flow increases, and finally, when the flow reaches a critical value, it couples to the upper mode. The coupled slow modes form two branches, and the gap between them continues to expand with further growing equilibrium flows (Figures 4d and e). Meanwhile, the two asymptotes to which the slow modes tend become closer until they merge with each other and cancel out, leaving behind only the lower branch of the coupled modes (Figure 4f). A further increase of the flow gradually causes this mode to disappear.

**Figure 5:**
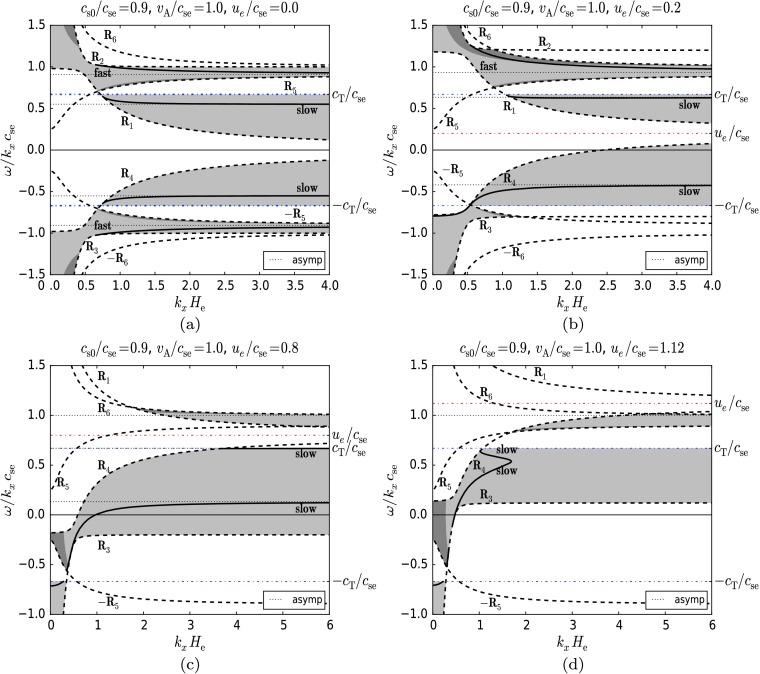
The dimensionless phase speed of MAG surface waves versus dimensionless horizontal wavenumber for $c_{\mathrm{s0}}/c_{\mathrm{se}}=0.9$, $v_{\mathrm{A}}/c_{\mathrm{se}}=1.0$ and (a) $u_{\mathrm{e}}/c_{\mathrm{se}}=0.0$, (b) $u_{\mathrm{e}}/c_{\mathrm{se}}=0.2$, (c) $u_{\mathrm{e}}/c_{\mathrm{se}}=0.8$, (d) $u_{\mathrm{e}}/c_{\mathrm{se}}=1.12$. The permitted regions of propagation for surface waves are shaded grey. The effect of increasing flow on the fast and slow magnetoacoustic-gravity modes can be seen. Note that as the permitted regions shift upwards, the backward-propagating fast mode vanishes (see panel (b)). The forward-propagating fast mode, while slowly shifting upwards together with the asymptote to which it tends, originates at increasingly higher wavenumbers (panels (c), (d)). The forward-propagating slow mode, while keeping its relatively constant position, is transferred to the permitted region containing the other slow mode (panel (c)). The backward-propagating slow mode continues to shift upwards (panel (b)), thus changes its direction of propagation (panel (c)) and gradually approaches the upper slow mode. Just before the asymptotes to which the slow modes tend reach each other, the slow modes couple and form two branches like in Figures 4d, e (this is not visible here). After the connection and cancellation of the asymptotes, only the lower branch of the coupled modes remains (panel (d)), which gradually disappears as the flow increases.

Figure 5 shows solutions of the dispersion relation (26) still taking $c_{\mathrm{s0}}/c_{\mathrm{se}}=0.9$, but with $v_{\mathrm{A}}/c_{\mathrm{se}}=1.0$ for four increasing values of the flow, $u_{\mathrm{e}}/c_{\mathrm{se}}$ starting from zero (Figures 5a – d). These parameters again describe a plasma that is slightly hotter at the upper part of the surface, similar to the case in Figure 4. However, the difference here is that the plasma-beta value is approximately 0.98 and the density ratio between the layers is 0.61. This situation is applicable to the region of the upper chromosphere. In case of these parameters, both the fast and slow MAG surface modes can propagate, as is visible in Figure 5a (which corresponds to the static case), both lying entirely within the light grey region ($A_{B}<0$) where the modes have declining vertical velocities in the upper medium ($z>0$).

The upward shift of the permitted regions and of the modes (as a result of the increasing flow) results in the disappearance of the backward-propagating fast mode (Figure 5b). The upper permitted region containing the forward-propagating fast mode is deformed as a result of the upward shift of the curve $R_{2}$. This causes the lower part of the mode to be transferred to a region (shaded dark grey) where the mode has a growing vertical velocity with height $z$ (Figure 5b). Next, at higher flow values, the mode, while slowly shifting upward together with the asymptote to which it tends, can only propagate at increasing wavenumbers (as shown by their absence in the range $0\le k_{x}H_{\mathrm{e}}\le6$ in Figures 5c and d).

The forward- and backward-propagating slow modes behave similarly to the case depicted in Figure 4. The forward propagating slow mode, whilst keeping its relatively constant position, is transferred to the permitted region containing the other slow mode (Figure 5c). Meanwhile, the backward-propagating slow mode carried away by the flow continues to shift upwards, and thus changes its direction of propagation (Figure 5c) and approaches the upper slow mode. Just before the asymptotes – to which the slow modes tend – reach each other, the slow modes couple and form two branches like in Figures 4d and e (this is not visible here). After the connection and cancellation of the asymptotes, only the lower branch of the coupled modes remains (Figure 5d), which gradually disappears as the flow increases.

**Figure 6:**
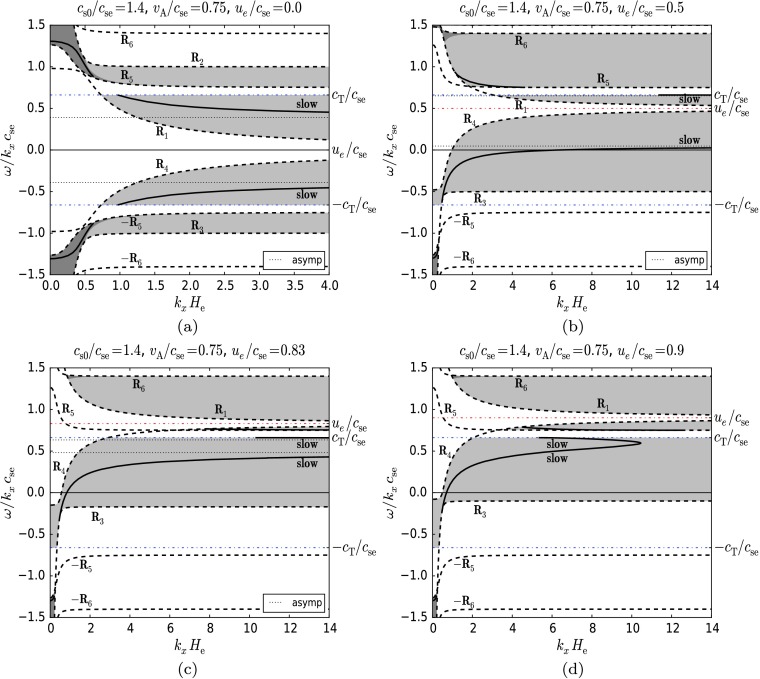
The dimensionless phase speed of surface MAG waves as a function of the dimensionless horizontal wavenumber for $c_{\mathrm{s0}}/c_{\mathrm {se}}=1.4$, $v_{\mathrm{A}}/c_{\mathrm{se}}=0.75$ and (a) $u_{\mathrm{e}}/c_{\mathrm{se}}=0.0$, (b) $u_{\mathrm {e}}/c_{\mathrm{se}}=0.5$, (c) $u_{\mathrm{e}}/c_{\mathrm{se}}=0.83$, (d) $u_{\mathrm{e}}/c_{\mathrm{se}}=0.9$. The permitted regions of propagation for surface waves are shaded grey. The effect of increasing flow on the slow magnetoacoustic-gravity mode can be seen. As the flow increases, the modes in the dark grey regions behave differently: the backward-propagating mode disappears, while its forward-propagating counterpart slips out from the dark grey region into the light grey region (see panel (b)) and later (at higher flow velocities) is transferred to the permitted region bounded by the cut-off curves $R_{4} \text{ and } R_{5}$ (panels (c) and (d)). The forward-propagating slow mode, while keeping its relatively constant position, is transferred to the permitted region containing the other slow mode (panel (c)). The backward-propagating slow mode continues to shift upwards (panel (b)), changes its direction of propagation (panel (c)), and approaches the upper slow mode. Finally, the asymptotes to which the modes tend reach each other and cancel out, and the modes couple to each other (panel (d)). The coupled modes gradually disappear as the flow increases.

Finally, in Figure 6 we show solutions of the dispersion relation (26) taking $c_{\mathrm{s0}}/c_{\mathrm{se}}=1.4$ and $v_{\mathrm{A}}/c_{\mathrm{se}}=0.75$. These parameters may model a situation where the upper plasma is hotter than the lower plasma. The plasma-beta value is approximately 4.18 and the density ratio is 0.41. We use these parameters for a direct comparison to the model used in Miles, Allen, and Roberts (1992). For these parameters only the slow MA mode may propagate in the limit of no gravity ($k_{x} H_{\mathrm{e}} \rightarrow\infty$). Thus, in Figure 6a (the static case), the slow MAG mode is visible. The mode is split into two parts. The upper curve (in the dark grey region) has a growing vertical velocity, while the lower part (in the light grey region) has a velocity decreasing in the $z>0$ region.

As the equilibrium flow increases, the modes in the dark grey regions behave differently: the backward-propagating mode disappears, while its forward-propagating counterpart slips out from the dark grey region into the light grey region (Figure 6b) and later (at higher flow velocities) is transferred to the permitted region bounded by the cut-off curves $R_{4}\text{ and } R_{5}$ (Figures 6c and d). The slow modes behave similarly to the previous cases: the backward-propagating slow mode shifts together with the flow (Figure 6b), changes its direction of propagation (Figure 6c), and finally reaches and couples to the forward propagating slow mode (Figure 6d). The coupled modes gradually disappear as the flow increases further.

It is of interest to deduce how the phase speed of a mode varies with the increasing flow magnitude at a specific wavenumber.

**Figure 7:**
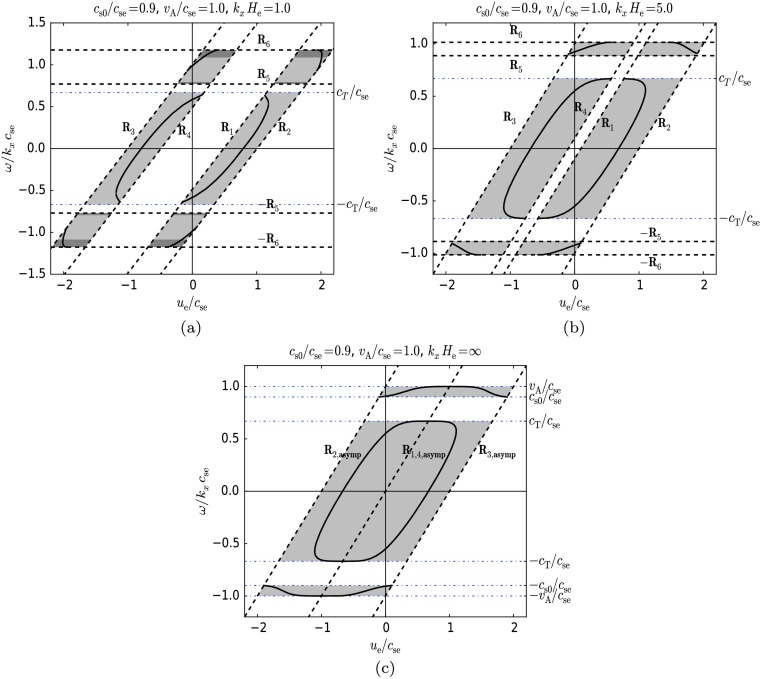
The variation of the dimensionless phase speed of surface MAG waves as a function of the dimensionless flow velocity at a given wavenumber for $c_{\mathrm{s0}}/c_{\mathrm{se}}=0.9$, $v_{\mathrm{A}}/c_{\mathrm{se}}=1.0$. Cases are: (a) $k_{x} H_{\mathrm{e}}=1.0$, (b) $k_{x} H_{\mathrm{e}}=5.0$, and (c) $k_{x} H_{\mathrm{e}}=\infty$. The permitted regions of propagation for surface waves are shaded grey. The effect of increasing the overall magnitude of the flow on the fast and slow magnetoacoustic-gravity modes can be seen. The modes shown here are the same fast and slow MAG surface modes as those displayed in Figure 5. The backward-propagating fast mode vanishes in all three cases. For a positive flow, between the $R_{1}$ and $R_{4}$ cut-off curves, there exists a non-permitted gap that causes in a break in the permitted flow velocity range for propagation at the forward-propagating fast and slow modes (see panels (a), (b)). The width of the gap decreases with increasing wavenumber and finally disappears in the $k_{x} H_{\mathrm{e}}=\infty$ limit (see panel (c)). Exactly the same occurs for ‘negative’ flows. The dispersion graph is rotated through $180^{\circ}$, and in this case, the backward-propagating slow and fast waves have a forbidden gap between the cut-off curves $R_{4}$ and $R_{1}$. As the positive flow increases, the phase speeds of forward-propagating fast and slow modes change little, by contrast the backward propagating slow mode is carried away by the flow and even changes its direction of propagation. The opposite is seen if the flow is decreased negatively, showing the symmetry of the problem. At a given flow velocity (positive and negative), the two slow modes couple, *i.e.* the Kelvin–Helmholtz instability occurs, and no asymptotic solutions exist any more.

In Figure 7 we show the variation of the dimensionless phase speed, $\omega/k_{x} c_{\mathrm{se}}$, of surface MAG waves as a function of the dimensionless flow velocity, $u_{\mathrm{e}}/c_{ \mathrm{se}}$. The parameters were set to $c_{\mathrm{s0}}/c_{ \mathrm{se}}=0.9$, $v_{\mathrm{A}}/c_{\mathrm{se}}=1.0$, and we chose three different values for the dimensionless wavenumber: (a) $k_{x} H_{\mathrm{e}}=1.0$, where the horizontal wavelength and the scale on which the (lower) equilibrium changes are comparable, (b) $k_{x} H_{\mathrm{e}}=5.0$, where the equilibrium varies on a scale lower than the wavelength and (c) $k_{x} H_{\mathrm{e}}=\infty$, where the length of a wave is far smaller than the change in equilibrium conditions, *i.e.* stratification is unimportant. From the choice of parameters the modes shown here are the same fast and slow MAG surface modes as those displayed in Figure 5. The permitted regions of propagation for surface waves are shaded grey. The $k_{x} H_{\mathrm{e}}=\infty$ case is equivalent to the gravity-free case, and therefore Figure 7c shows the behaviour of the fast and slow MAG surface modes that correspond to the asymptotes of the modes in Figure 5.

It is visible in all three figures that as the flow starts to increase from zero, the phase speeds of all modes increase. The backward-propagating fast mode vanishes in all three cases. A forbidden gap arises, where surface modes are not allowed to propagate, bounded by the $R_{1}$ and $R_{4}$ cut-off curves. This causes the forward-propagating fast and slow modes to exist in two separate flow intervals below and above the gap (Figures 7a, b). However, with increasing wavenumber, the width of the gap decreases, eventually disappearing in the $k_{x} H_{\mathrm{e}}\to\infty$ limit (Figure 7c). See Equations (54) – (56) for the asymptotic values of the cut-off curves $R_{1}$, $R_{2}$, $R_{3}$, $R_{4}$, $R_{5}$, $\text{and } R_{6}$.

The phase speeds of the forward-propagating fast and slow modes clearly change little with flow speed. For lower flow velocities they increase, and for higher flow velocities they decrease with growing flow. On the other hand, the phase speed of the backward-propagating slow mode changes substantially with flow speed, being carried away by the flow (see the parts in Figure 7 where the phase speed increases together with the flow with unit slope). It is also easily observable that the backward-propagating slow mode changes the direction of propagation and couples to the other slow mode.

For flows in the negative direction, the opposite behaviour occurs. The forward-propagating slow modes are decelerated by this counter-flow and even reverse their direction of propagation. The backward-propagating fast and slow mode phase speeds are barely changed by the increase in the magnitude of the negative flow. This is due to the property of the dispersion relation (26) that it is symmetric for the Doppler shifted frequency ($\Omega$) in the lower layer part.

## Conclusion

We study MAG surface wave propagation with the consideration of a steady uniform flow (in the horizontal $x$-direction) in the bottom layer of two semi-infinite plasmas under gravity described by the fully static equilibrium given in Miles and Roberts (1992). The equilibrium leads to a velocity shear, with the bottom layer in relative motion to the fixed frame of reference of the upper magnetic layer. It is found that this consideration of flow has important consequences on the propagation of MAG surface waves.

With respect to the propagation of surface waves, the flow is shown to have an effect in some distinct ways. By increasing the flow parameter $u_{\mathrm{e}}/c_{\mathrm{se}}$ from zero upwards, the cut-offs for determining regions of surface wave propagation in the $\omega/k_{x}$–$k_{x}$ plane are shifted. The disappearance of the fast wave can be accounted for by this shift, in some cases (see, *e.g.* Figure 4a). Another aspect of the consideration of bulk background flow is the change in the phase speeds of the surface waves. Figures 4–6 show that the backward-propagating solution is decelerated as the flow is increased. At a wavelength threshold, it can eventually become a forward-propagating wave in the static frame of reference. We clearly observe this behaviour in Figure 7. Here the flow is increased, with the wavenumber remaining constant, and the solutions clearly cross the axis to become forward propagating. The change in direction of propagation of the surface waves has been shown to lead to the occurrence of negative-energy waves, which may be of interest in energy transfer between waves of positive and negative energy (Ryutova 1988), resonant absorption (Ruderman and Wright 1998, Tirry *et al.*, 1998, Erdélyi and Taroyan, 2003), or explosive instabilities (Joarder, Nakariakov, and Roberts, 1997). As the flow velocity shear between the two layers is increased, eventually a point is reached where the Kelvin–Helmholtz instability appears (see *e.g.* Chandrasekhar 1961); the two wave solutions couple together. This is demonstrated in Figures 4–7, where the solutions approach one another as the flow increases, becoming complex conjugates, respectively. This means that both have the same real phase speed, but one wave has a positive imaginary part and is therefore over-stable with increasing amplitude in time, whilst the other wave has a negative imaginary part and is therefore damped over time.

Meridional flows of the interior of the Sun are very slow, up to $0.2~\mbox{km}\,\mbox{s}^{-1}$. We take this value as $u_{\mathrm{e}}$ and the sound speed as $7~\mbox{km}\,\mbox{s}^{-1}$ such that $u_{\mathrm{e}}/c_{\mathrm{se}}=0.03$ for the model proposed for Figure 4. It is clear from this value that this type of velocity shear will only have a modest effect on the propagation of MAG surface waves in the proposed model. However, this type of velocity shear is not the only possible shear for the model described in Figure 4. Velocity shears between the two layers could also be attributed to differential rotation of the Sun. If we take a high differential rotation velocity of $2.0~\mbox{km}\,\mbox{s}^{-1}$ i.e. $u_{\mathrm{e}}/c_{\mathrm{se}}=0.29$, then the ratio is higher, but as shown in Figure 4, the flow speed is not high enough for backward-propagating waves to reverse their direction. Therefore no negative energy waves or Kelvin–Helmholtz instability will occur.

Evershed flows in sunspots can reach up to $6~\mbox{km}\,\mbox{s}^{-1}$. Applying this to Figure 5 and taking $c_{\mathrm{se}}=7~\mbox{km}\,\mbox{s}^{-1}$ and $u_{\mathrm{e}}=6~\mbox{km}\,\mbox{s}^{-1}$ and thus $u_{\mathrm{e}}/c_{\mathrm{se}}=0.86$, it can be seen from Figures 5 and 7 that this flow speed is high enough for the backward-propagating waves to reverse their direction of propagation. Thus some running penumbral waves (RPW) may become negative energy waves. This could also have implications for interpreting the observations of RPW, as some may have very slow phase speeds because they are backward-propagating waves.
